# Retraction Note: Melatonin loaded poly(lactic-co-glycolic acid) (PLGA) nanoparticles reduce inflammation, inhibit apoptosis and protect rat’s liver from the hazardous effects of CCL4

**DOI:** 10.1038/s41598-026-69231-w

**Published:** 2026-09-02

**Authors:** Alyaa Farid, Valina Michael, Gehan Safwat

**Affiliations:** 1 Biotechnology Department, Faculty of Science, Cairo University, Giza, Egypt; 2 Faculty of Biotechnology, October University for Modern Sciences and Arts (MSA), Giza, Egypt

Retraction of: https://doi.org/10.1038/s41598-023-43546-4, published online 30 September 2023

The Editors have retracted this Article.

After publication, concerns were raised regarding the data presented in the Figures, specifically:

- Figs. 2A, 9 and 10 appear to have repetitive features in the microscopy images;

- Fig. 9C and G appear to overlap with rotation;

- Fig. 10D, E and H appear to originate from the same sample.

Additionally, the error bars in Figs. 4–8 appear unusually consistent and proportional to the means.

The Authors have not provided the underlying raw microscopy data upon request. The Editors therefore no longer have confidence in the preseted data.

Alyaa Farid does not agree with this retraction. Valina Michael and Gehan Safwat have not responded to any correspondence from the Editors about this retraction.

